# Relationship of Circulating CXCR4^+^ EPC with Prognosis of Mild Traumatic Brain Injury Patients

**DOI:** 10.14336/AD.2016.0610

**Published:** 2017-02-01

**Authors:** Yunpeng Lin, Lan Lan Luo, Jian Sun, Weiwei Gao, Ye Tian, Eugene Park, Andrew Baker, Jieli Chen, Rongcai Jiang, Jianning Zhang

**Affiliations:** ^1^Department of Neurosurgery, Tianjin Medical University General Hospital, Tianjin Neurological Institute, Key Laboratory of Post-neurotrauma Neuro-repair and Regeneration in Central Nervous System, Ministry of Education and Tianjin City, Tianjin 300052, China; ^2^Department off Psychological Science, Tianjin Medical University General Hospital, Tianjin 300052, China; ^3^Department of Traumatic Critical Care Unit, St. Michael’s Hospital, Toronto, Canada; ^4^Department of Neurology, Henry Ford Hospital, Detroit, MI USA; ^5^Department of Geriatrics, Tianjin Geriatrics Institute, Tianjin Medical University General Hospital, Tianjin, China

**Keywords:** traumatic brain injury, endothelial progenitor cells, C-X-C chemokine receptor type 4 (CXCR-4), stromal derived factor-1 (SDF-1)

## Abstract

To investigate the changes of circulating endothelial progenitor cells (EPCs) and stromal cell-derived factor-1α (SDF-1α)/CXCR4 expression in patients with mild traumatic brain injury (TBI) and the correlation between EPC level and the prognosis of mild TBI. 72 TBI patients (57 mild TBI, 15 moderate TBI patients) and 25 healthy subjects (control) were included. The number of circulating EPCs, CD34^+^, and CD133^+^ cells and the percentage of CXCR4^+^ cells in each cell population at 1,4,7,14,21 days after TBI were counted by flow cytometer. SDF-1α levels in serum were detected by ELISA assay. The patients were divided into poor and good prognosis groups based on Extended Glasgow Outcome Scale and Activity of Daily Living Scale at 3 months after TBI. Correlation analysis between each detected index and prognosis of mild TBI was performed. Moderate TBI patients have higher levels of SDF-1α and CXCR4 expression than mild TBI patients (P < 0.05). The percentage of CXCR4^+^ EPCs at day 7 post-TBI was significantly higher in mild TBI patients with poor prognosis than the ones with good prognosis (P < 0.05). HAMA and HAMD scores in mild TBI patients were significantly lower than moderate TBI patients (P < 0.05) in early term. The percentage of CXCR4^+^ EPCs at day 7 after TBI was significantly correlated with the prognosis outcome at 3 months. The mobilization of circulating EPCs can be induced in mild TBI. The expression of CXCR4^+^ in EPCs at 7 days after TBI reflects the short-term prognosis of brain injury, and could be a potential biological marker for prognosis prediction of mild TBI.

Traumatic brain injury (TBI) is a complex injury with a broad spectrum of symptoms and disabilities [1, 2]. Approximately 75% of all TBIs are mild [3, 4]. In last 20 years, with the increasing number of military conflicts, the incidence of mild TBI caused primarily by blast head injury is sharply increased, and up to 49.7% of American ex-servicemen with blast head injury develop mild TBI [5]. This has resulted in more attention has been paid to mild TBI [6].

Tanriverdi et al [7] have demonstrated that headache, dizziness and memory impairment are the most common symptoms of mild TBI which affect patient’s mental and physical health and thereby influence a patient’s daily life and job. However, mild TBI can be easily missed due to its mild symptoms and lack of long-term follow-up, and because of this it is often not treated effectively. Early identification of TBI can help guide treatment and provide reference information for later rehabilitation, facilitating enhancement in therapeutic efficacy and rational assignment of medical treatment resources. To the best of our knowledge, no biological markers have yet been identified for the early prognosis of mild TBI, and searching for such biological markers has become one of the most concerned topics in the field of TBI research.

Endothelial progenitor cells (EPCs) play a critical role in maintaining the integrity of vascular endothelium, participate in post-traumatic angiogenesis and tissue repair, and show encouraging application prospects [8, 9]. Previous studies have demonstrated that circulating EPC numbers may be a cytological index for predicting the prognosis of severe TBI patients [10, 11]. Our previous study further confirmed that EPCs were rapidly mobilized from the bone marrow into the blood after TBI, and recruited to sites of injury, where they promoted neovascularization, or directly proliferated and differentiated into endothelial cells to form vessels, participate in tissue repair and thereby improve neural functional outcome [12]. Lower levels of circulating EPCs are correlated with worse functional outcome in severe TBI patients [12]. However, how circulating EPC levels change in mild TBI is poorly understood.

Stromal cell-derived factor-1 (SDF-1), i.e., chemokine (C-X-C motif) ligand 12 (CXCL12), belongs to the CXC chemokine family [13]. Its receptor, chemokine (C-X-C motif) receptor type 4 (CXCR-4) can be expressed on the surface of EPCs [14], monocytes, lymphocytes, hemopoietic stem cells/progenitor cells [15-17] neurons and glial cells [18]. Petit et al found that by binding to CXCR4, SDF-1α promotes EPC migration along a SDF-1α concentration gradient, and inhibits the apoptosis of EPCs [19]. Li et al [20] have demonstrated that SDF-1α mobilizes CD34^+^/CXCR4^+^ cells to the sites of injury after TBI, which promotes neovascularization in the traumatic region and benefits for post-traumatic brain tissue repair and functional reconstruction. Therefore, the SDF-1α/CXCR4 axis may play an important role in the repair of neurovasculature after TBI. It is hypothesized that (1) characteristic changes of peripheral blood EPCs also exist in mild TBI patients; (2) peripheral blood SDF-1α and the CXCR4 expression in different cell populations might be related to the prognosis of mild TBI patients.

## MATERIALS AND METHODS

### General data

TBI patients admitted to Tianjin Medical University General Hospital, China from May 2012 to April 2015 and aged 16-75 years old were screened. The study protocol was approved by the Institutional Review Board of Tianjin Medical University, China for the use of human subjects in biomedical research. All subjects or guardians signed consent forms before enrollment. All patients were admitted in the department of neurosurgery within 12h after injury, and received a complete neurological and imaging exam.

#### Exclusion criteria

Patients with open TBI or with complex trauma involving body limbs and trunk, hematologic disorders, cancer and patients on sedation were excluded. Severe TBI patients with Glasgow Coma Scale (GCS) <9 or patients that could not tolerate conservative treatment were excluded.

#### Inclusion criteria

Closed TBI patients with GCS between 9 and 15 were included. TBI patients with a GCS of 13~15 were assigned to a mild TBI group, while those with a GCS of 9~12 were enrolled as the moderate TBI group. Simultaneously, 25 age and gender-matched healthy volunteers were recruited as the control group. Patients enrolled were treated according to the guidelines for TBI management [21]. All patients have imaging evidence of acute trauma-related intracranial lesion in the emergency department by computed tomography (CT) scans such as subarachnoid hemorrhage (SAH), cerebral contusion, intracranial hematomas and so on. All the TBI patients enrolled were conservatively treated. The following treatments were provided for all TBI patients as needed, including osmotic dehydration treatment using mannitol if the intracranial pressure was diagnosed to increase, glucose-lowering treatment for hyperglycemia, antihypertensive treatment for hypertension, acid inhibitor for peptic ulcer, supportive therapy for nutrition, fluid, and electrolyte balance requirements.

### Collection of blood samples

At 7:00 a.m. of days 1, 4, 7, 14 and 21 after TBI, 6 mL of peripheral blood was collected from the TBI patients and 25 normal controls. 4 mL of the blood sample was added to an ethylene diamine tetraacetic acid (EDTA)-K2 anticoagulant tube. Peripheral blood mononuclear cells (PBMCs) were isolated and subjected to flow cytometry (BD FACS Calibur, BD Biosciences, San Jose, CA). The remaining 2 mL of blood was centrifuged at 3,000 r/min for 15 minutes in a coagulation-promoting vacuum tube and the supernatant was collected to test for SDF-1α.

Blood from the normal controls was used to establish the baseline for flow cytometry and ELISA assay.

### Flow cytometry

2 mL of the EDTA blood sample was centrifuged at 300 ×g for 20 minutes at room temperature. The isolated cells were washed with phosphate buffer saline (PBS) (pH 7.2) three times, re-suspended in 200μL PBS supplemented with 2 mmol/L EDTA and 0.5% bovine serum albumin (BSA). Thereafter, cells were labeled with fluorescein isothiocyanate-conjugated CD34 monoclonal antibody (BD Pharmingen, San Jose, CA, USA), R-phycoerythrin-conjugated monoclonal CD133 antibody (Miltenyi Biotech, Gladbach, Germany) and allophycocyanin-conjugated monoclonal CD184 (CXCR4, BD Pharmingen) for 20 minutes at room temperature. Stained cells were washed with PBS/BSA and then analyzed by flow cytometry. Three isotype controls of R-phycoerythrin-, fluorescein isothiocyanate- and allophycocyanin- conjugated mouse IgG were used for background nonspecific binding.

**Figure 1.**
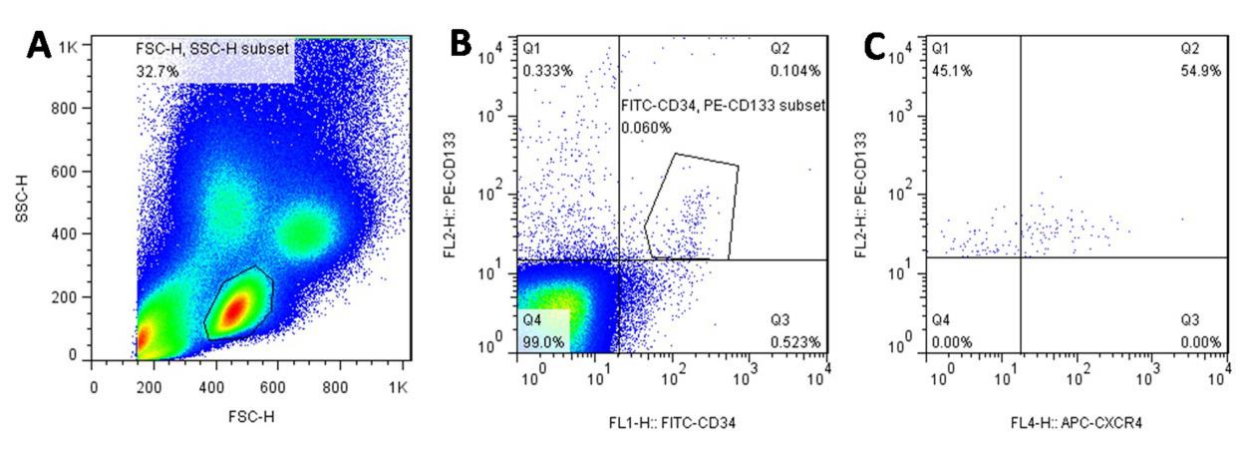
**A sample illustration of detecting the percentage of CXCR4^+^ cells in circulating EPCs. (A)** Cells were first run on a forward and side scatter to select mononuclear cells. **(B)** The selected cells were then gated on FITC-CD34 and PE-CD133 to choose CD34^+^, CD133^+^ and double positive EPCs. (C) The percentages of CXCR4^+^ cells on each cell population (EPCs) were finally measured by APC-CXCR4 staining.

Cells were first run on forward scatter and side scatter to select mononuclear cells to reduce signal noises from cell aggregates, platelets, and cellular debris. 8×10^6^ CD34^+^, CD133^+^ and CD34^+^/CD133^+^ cells were counted. The percentage of CXCR4^+^ cells in each cell population was calculated. Selection of cell population by flow cytometry is shown in Fig. 1. Comparisons between groups were then performed.

### Serum SDF-1α measurement by ELISA

100 μg of each cell standard sample was added to an ELISA reader and 100 μL distilled water was used in the control group. With the exception of control group, 50μL enzymatic labeling solution was added. All ELISA plates were incubated at 37? for 1 hour, then thoroughly washed, and dried using absorbent paper. This procedure was repeated three times. Then chromogenic agents A, B (each 50 μL) were added to each well for a 15 min reaction in the dark at room temperature. 50 μL stop buffer was added to each well to terminate the reaction. The absorbance at 450 nm was read. According to standard curve, serum level of SDF-1α was calculated.

### Prognosis and psychological outcome follow-ups

#### 1. GCS and grouping

Patients were assigned to mild TBI group and moderate TBI group according to GCS on admission. The TBI patients with GCS of 13~15 were assigned to the mild TBI group; those with a GCS of 9~12 were enrolled as moderate the TBI group. The mild TBI patients were further assigned to either good or poor prognosis groups on the basis of 3-month follow up outcomes.

Prognosis post TBI was evaluated using the Extended Glasgow Outcome Scale (GOS-E) and Activity of Daily Living Scale (ADLS) [22]. The GOS-E, derived from the GOS, is a 5-point scale, with higher points indicating better prognosis. Compared to the GOS, the GOS-E is more sensitive to changes in TBI, even to slight symptoms (headache or dizziness). The ADLS was composed of the Physical Self-Maintenance Scale (PSMS) and Instrumental Activities of Daily Living Scale (IADL). The IADL provides detailed scores and can help effectively evaluate the prognosis of TBI patients. The PSMS contains six items and the IADL contains 8 items, with 1-4 points per item. A score of 14 points is considered normal, and score of less than 14 points suggests different degrees of function impairment.

#### Group A (Good prognosis group)

TBI patients with full mark of GOS-E and ADLS. (GOS-E score of 8; ADLS score of 14 points)

#### Group B (Poor prognosis group)

TBI patients without full mark of GOS-E and ADLS. (GOS-E score ≤7 points; ADLS score ≥15 points)

#### 2. Psychological outcome measurements

The Hamilton Anxiety Scale (HAMA) and Hamilton Depression Scale (HAMD) were employed to gauge the possible anxiety and depression symptoms of TBI patients at 3 and 6 months after TBI. The HAMA (14 items,scores in the range of 0-56) were used to evaluate the level of anxiety (0-17 indicative of mild anxiety, 18 - 24 mild to moderate anxiety, 25 - 56 moderate to severe anxiety). The HAMD (17 items,scores in the range of 0-51) were used in this study to measure the severity of depression (normal 0-7, mild depression 8-13, moderate depression 14-18, severe depression 19-22, very depression>=23). These two scales were used in this study to gauge the severity of anxiety and depression symptoms in TBI patients.

### Statistical analysis

All data were statistically processed using SPSS 17.0 software (SPSS, Chicago, IL, USA). The Shapiro-Wilk test was used in each group to evaluate whether data have a normal distribution. The normally distributed measurement data were expressed as the mean ± SD. The unpaired t-test and chi-square test were used. P < 0.05 was considered statistically significant. Stepwise binary logistic regression was used (inclusion criteria α = 0.05, exclusion criteria α = 0.10) to screen the main factors that influence the prognosis of TBI patients. Pearson’s correlation coefficient was used to indicate the correlation between detected indices and the prognosis of TBI. The receiver operating characteristic (ROC) curve was used to assess the accuracy of predictions.

## RESULTS

### General data of TBI patients (Table 1)

A total of 72 TBI patients (57 mild TBI, 15 moderate TBI patients) and 25 normal controls were enrolled. With the exception of hospital days and GCS scores at admission, there were no significant differences in gender, age, time interval between onset and hospital admission and injury causes between mild and moderate TBI patients.

**Table 1:** Comparison of general data of TBI patients.

Item	Mild TBI group (n = 57)	Moderate TBI group (n = 15)	*P* value
Gender [n (%)]			0.793
Male	40 (70.18%)	10 (66.67%)	
Female	17 (29.82%)	5 (33.33%)	
Age (year)	48.19±16.63	41.80±19.58	0.283
Time interval between onset and hospital admission (hour)	5.34±2.16	5.10±2.69	0.758
Hospital days	13.87±7.14	24.60±7.85	0.000*
GCS score at admission	14.44±0.60	10.47±0.92	0.000*
Open/closed TBI [n (%)]	15 (26.3%)/42(73.7%)	4 (26.7%)/11(73.3%)	0.607
Injury causes [n (%)]			
Stumble	12 (21.05%)	0 (0%)	0.052
Violence	14 (24.56%)	3 (20%)	0.711
Motor vehicle accidents	21 (36.84%)	9 (60%)	0.106
High falls	10 (17.54%)	3 (20%)	0.826

All data were expressed as the mean ± SD.

* *P* < 0.05. TBI: Traumatic brain injury; GCS-E: The Extended Glasgow Outcome Scale.

**Figure 2.**
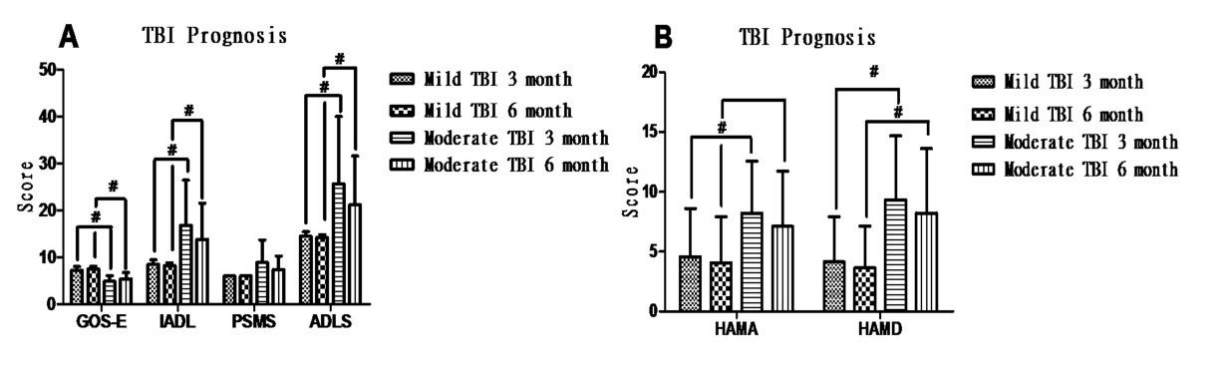
**Prognosis and mental state of TBI patients. (A)** Moderate TBI significantly induces a worse prognosis after TBI compared to the mild TBI group at 3 and 6 months after TBI, identified by GOS-E, IADL and ADLS scores. **(B)** HAMA and HAMD scores were significantly increased in the moderate TBI group compared to mild TBI group (*p* < 0.05) at 3 and 6 months after TBI. There was no significant difference in HAMA and HAMD scores between 3 and 6 months after discharge in mild or moderate TBI patients, respectively.

Taking the measured indices of the normal control (n = 25) as a baseline level, indexes comparison among mild TBI, moderate TBI and control groups were performed. A previous study has reported that the majority of symptoms could be relieved 1-3 months after TBI [23]. Mild TBI patients with GOS-E=8 and ADLS =14 were designated as good prognosis, while the ones with GOS-E less than 8 or ADLS more than 14 were set as poor prognosis. At the 3-month follow up, 27 mild TBI patients had good prognosis (47.4% of mild TBI patients) and 30 patients from the mild group had a poor prognosis. Based the above prognosis standard, none of moderate TBI patients in this study had a good prognosis, three of the moderate TBI patients had aggravation 7 days after admission with GCS dropping to 6-8. The prognosis of TBI patients at different time points was shown in Fig. 2. There was no significant difference in each scale score between 3 month and 6 month after TBI.

**Figure 3.**
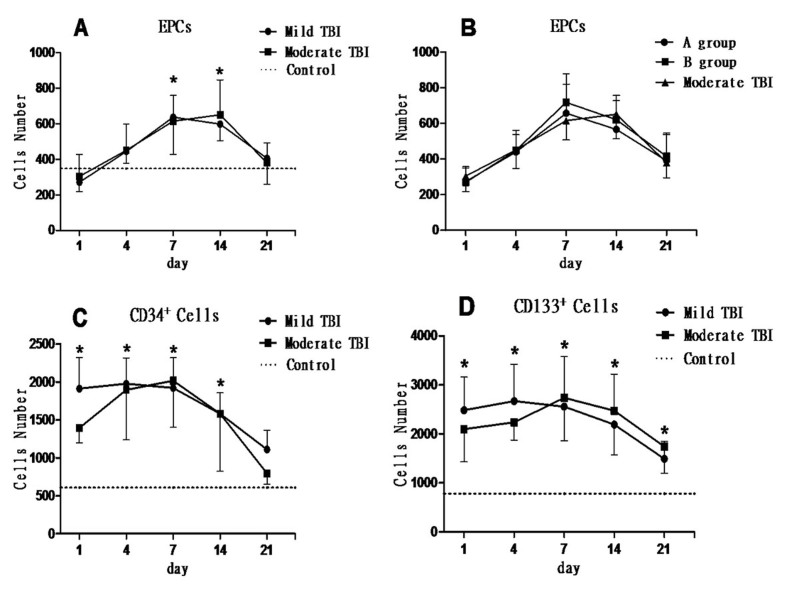
**Number of CD34^+^, CD133^+^ and EPCs in the peripheral blood. (A)** Number of circulating EPCs in mild and moderate traumatic brain injury (TBI) patients, showing a similar tendency of “from low to high”, peaked at 7 days and then gradually decreased and was significantly higher than that in the control group at 7 and 14 days after TBI (*p < 0.05). **(B)** Mild TBI patients were further divided into a good prognosis group (group A) and a poor prognosis group (group B). There was no significant difference in EPC number among group A, group B and moderate TBI group (*P* > 0.05). **(C)** CD34^+^ cell number in the peripheral blood of mild and moderate TBI patients was very high in the early stage after TBI and began to significantly decrease at 7 days after TBI, and was significantly higher than control group at 1, 4, 7 and 14 days after TBI (*p < 0.05). There was no significant difference in CD34^+^ cell number between mild and moderate TBI groups (*p* > 0.05). (D) CD133^+^ cell number in the peripheral blood of all TBI patients was also very high in the early stage after TBI and began to significantly decrease at 7 days after TBI, and was significantly higher than the control group at 1, 4, 7, 14 and 21 days after TBI (*p < 0.05). There was no significant difference in CD133^+^ cell number between mild and moderate TBI group (*p* > 0.05).

### The changes of circulating EPCs, CD34^+^ and CD133^+^ after TBI:

Fig. 1 shows a typical example of EPCs cells that were labeled with both CD133 and CD34 antibodies. We also included triple labeled cells (antibodies for CXCR4, CD34 and CD133) were used to estimate the numbers of CXCR4+ EPCs. Early stage EPCs (marked by CD133) and total endothelial cells (marked by CD34) were also measured by flow cytometry.

Compared with the normal control, circulating EPC levels from either mild or moderate TBI patients were slightly decreased at one day after TBI, then rapidly increased, peaked at 7 to 14 days after TBI and then gradually decreased to baseline level (7^th^ day: F = 6.038, P = 0.017; 14^th^ day: F = 7.639, *P* = 0.009). However, the levels of circulating EPCs either between mild and moderate TBI or between mild TBI patients with good (group A) and bad prognosis (group B) did not show significant difference (*P* > 0.05) (Fig. 3A and 3B).

**Figure 4.**
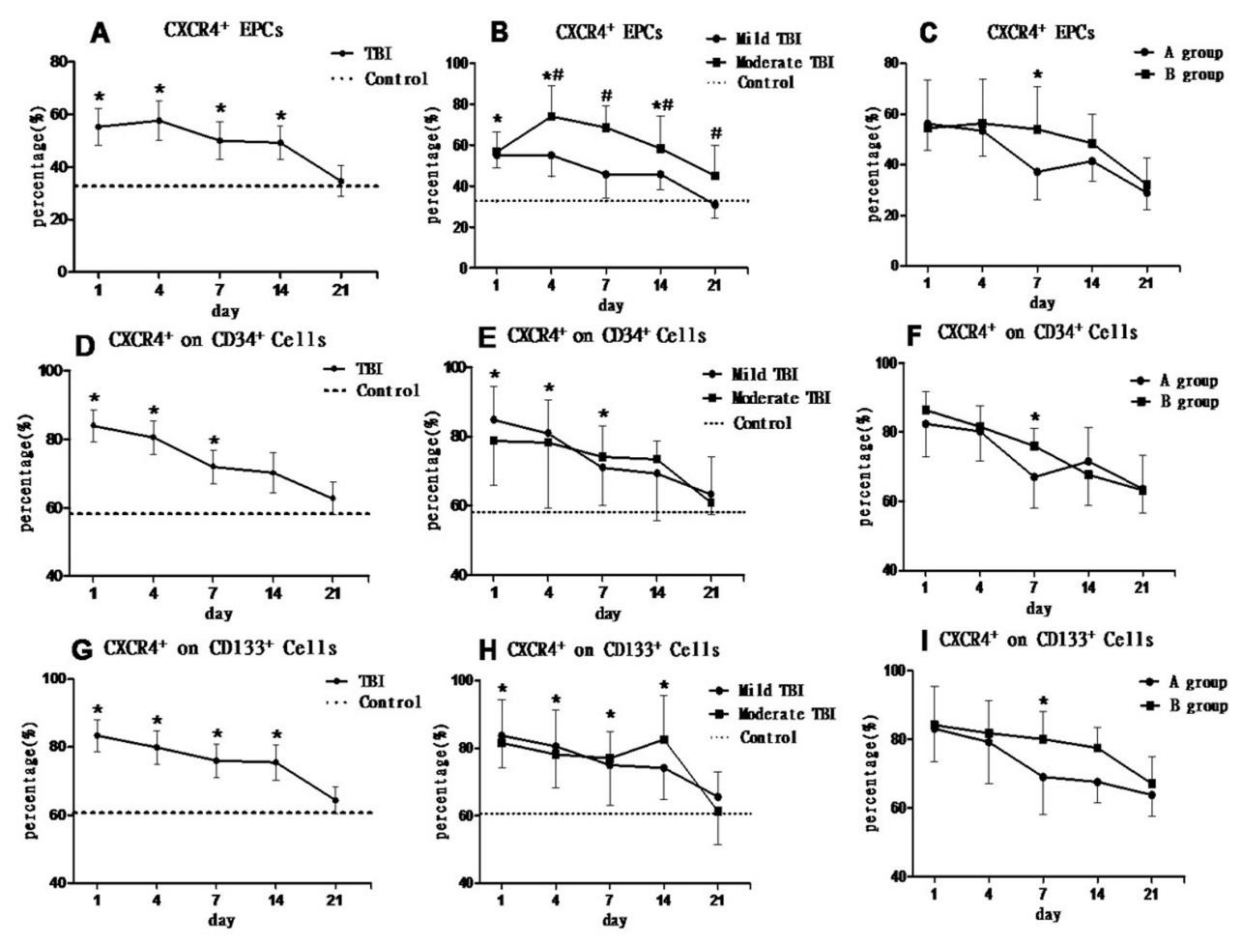
**Percentage of CXCR4^+^ cells on EPCs, CD34^+^ and CD133^+^ cells in TBI patients. (A, D, G)** show the percentages of CXCR4+ cells in EPCs, CD34^+^ or CD133^+^ cells were significantly higher than the baseline level (control group; *p < 0.05) at early stage (within 14 days) after TBI and then gradually decreased in the following days. **(B)** The percentage of CXCR4^+^ cells in EPCs of mild TBI patients was significantly higher than that in the control group (*p < 0.05) at 1, 4, and 14 days after TBI. The percentage of CXCR4^+^ cells in EPCs in the mild TBI group was significantly lower than the moderate TBI group at 4, 7, 14, 21 days (^#^*p* < 0.05). **(E, H)** There was no significant difference between mild TBI and moderate TBI groups (*P* > 0.05) in circulating CD34^+^ and CD133^+^ cell expression. **(C, F, I)** Mild TBI patients were further divided into a good prognosis group (group A) and a poor prognosis group (group B). **(C)** The percentage of CXCR4^+^/EPC in group A was significantly lower than group B at 7 days after TBI (F = 11.375, *p = 0.002). **(F)** The percentage of CXCR4^+^/CD34^+^ cells in group A was significantly lower than group B at 7 days after TBI (F = 6.124, *p = 0.02). (I) The percentage of CXCR4^+^/CD133^+^ cells in group A was significantly lower than group B at 7 days after TBI (F = 7.435, *p = 0.011).

After TBI, both CD34^+^ and CD133^+^ cells were significantly increased within 14 days (*p* < 0.05, Fig. 3-C-D) and gradually decreased to baseline level at 21 days when compared to normal controls (*p* < 0.05). There were no significant differences in the level of circulating CD34^+^ and CD133^+^ cells between mild TBI and moderate TBI patients or between mild TBI patients with good (group A) and bad prognosis (group B) (*p* > 0.05) (Fig. 3C, D).

### The changes of circulating CXCR4^+^/EPC, CXCR4^+^/CD34^+^ and CXCR4^+^/CD133^+^ after TBI

We further tested the changes of CXCR4 expression in EPCs, CD34^+^ or CD133^+^ cells in mild and moderate TBI patients. The percentage of CXCR4^+^ cells in EPCs was significantly increased at an early stage (within 14 days) after TBI compared to the normal control. The level of CXCR4^+^/EPC gradually decreased to baseline at 21 days after TBI. The percentage of CXCR4^+^/EPCs was significantly decreased in mild TBI patients compared to the moderate TBI groups during 4-21 days after TBI (*p* < 0.05). The patients with a mild TBI and good prognosis (group A) had lower CXCR4^+^/EPCs levels 7 days after TBI (F = 11.375, *p* = 0.002) than the patients with mild TBI with poor prognosis (group B), (Fig. 4A, B, C).

The changes in the level of CXCR4^+^/CD34^+^ and CXCR4^+^/CD133^+^ cells were similar to the changes of CXCR4^+^/EPCs after TBI. However, there was no significant difference in the levels of either CXCR4^+^/CD34^+^ or CXCR4^+^/CD133^+^ cells between mild TBI and moderate TBI patients (*p* > 0.05) (Fig. 4E, H). However, the percentage of CXCR4^+^/CD34^+^ and CXCR4^+^/CD133^+^ cells were significantly decreased in mild TBI with good prognosis (group A) when compared to the one with bad prognosis (group B) at day 7 after TBI (for CXCR4^+^/CD34^+^: F = 6.124, P = 0.02; for CXCR4^+^/CD133^+^: F = 7.435, *p* = 0.011) (Fig. 4F-I).

### Serum SDF-1α level

**Figure 5.**
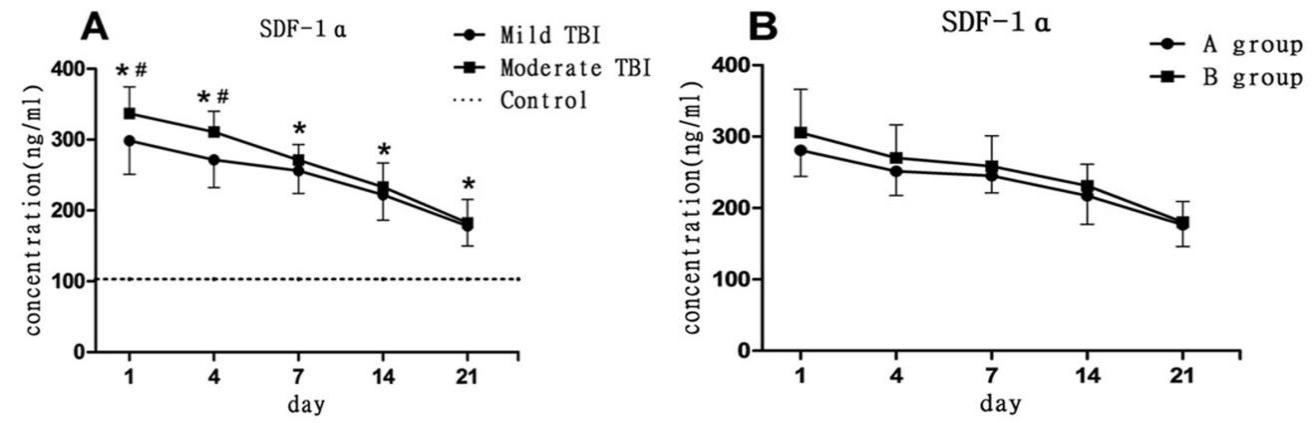
**Changes in serum SDF-1α level after TBI. (A)** Serum SDF-1α levels in mild and moderate TBI groups were significantly increased at 1,4,7,14 and 21days after TBI compared to control group (*p < 0.05). SDF-1 was significantly higher in the moderate TBI group than the mild TBI group at 1 and 4 days after TBI (#*p* < 0.05). **(B)** Mild TBI patients were further divided into a good prognosis group (group A) and a poor prognosis group (group B). There was no statistical significance in SDF-1α levels between group A and B.

SDF-1α is released from injured brain tissue to peripheral blood after TBI [24]. The SDF-1α ELISA assay determined that the serum SDF-1α level significantly increased immediately after TBI and then gradually decreased (Fig. 5). SDF-1α levels were significantly higher in the moderate TBI patients than in the mild TBI patients (*p* < 0.05) at 1 and 4 days after TBI. However, there is no significant differences in serum SDF-1α levels between the mild TBI with good progress (group A) and those with poor progress (group B) (*p* > 0.05).

**Figure 6.**
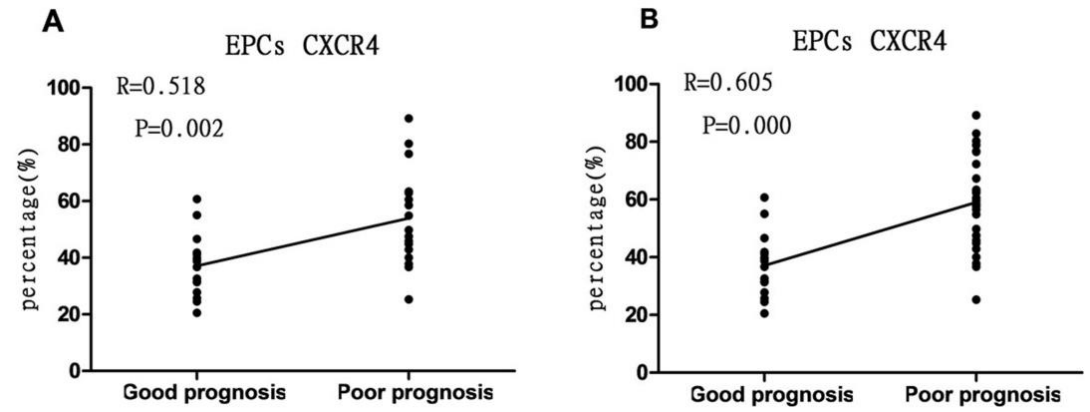
**Correlation analysis. (A)** There is significant relationship between percentage of CXCR4^+^/EPCs (7 day after admission) in mild TBI patients (R= 0.518, *p* = 0.002) with the prognosis at 3 months after TBI. **(B)** There is significant correlation between percentage of CXCR4^+^/EPCs (7 day after admission) in all TBI patients (R= 0.605, *p* = 0.000) with the prognosis at 3^rd^ month after TBI.

### Follow-up outcomes

57 mild TBI patients and 15 moderate TBI patients were included in the follow-up analysis. The prognosis of TBI patients is shown in Fig. 2 and 6.

#### Anxiety measurements by HAMA scores

Among mild TBI patients, eight presented with anxiety 3 months after TBI (14.0% of mild TBI patients). According to HAMA scores, two of them had moderate anxiety and six had mild anxiety. Among the moderate TBI patients, four presented different degrees of anxiety (26.7% of moderate TBI). Two of them had moderate anxiety and the other two had mild anxiety. Compared with moderate TBI patients, those with mild TBI had lower HAMA score (less anxiety) at 3 months (*P* < 0.05), but not at 6 months (*P* > 0.05) after TBI.

#### Depression measurements by HAMD scores

At 3 months after TBI, five mild TBI patients had symptoms of depression. One of them had moderate depression and four of them had mild depression. Three moderate TBI patients suffered from depression, where two were moderate and one had mild depression. Compared with the moderate TBI patients, the mild ones had lower HAMD scores (lower depression) at 3 and 6 months after TBI (*P* < 0.05). These findings suggested that moderate TBI patients had more severe anxiety and depression than mild ones.

### Correlation between percentage of CXCR4^+^ cells in each cell population and TBI patient’s prognosis

Taking the percentage of CXCR4^+^ cells in peripheral blood CD34^+^, CD133^+^ cells and EPCs as a concomitant variable and patient’s prognosis at 3 months as a dependent variable, binary logistic regression was performed (Table 2). Among all concomitant variables, only X3, i.e., the percentage of CXCR^+^/EPCs at 7 days after TBI was significant (P = 0.036, OR= 1.135) and was taken as an independent risk factor influencing the prognosis of mild TBI patients. The logistic regression equation of X3 was LogitP=1.135X_3_-23.963. The other variables were not significant (*P* > 0.05) (Table 2).

**Table 2:** Logistic regression analysis results of each factor that influences the prognosis of patients with mild traumatic brain injury

Concomitant variable	Regression coefficient	Standard error	Wald value	*p*	OR	95% confidence interval	
						Lower limit	Upper limit
X_1_	0.149	0.077	3.789	0.052	1.161	0.999	1.350
X_2_	0.108	0.059	3.407	0.065	1.114	0.993	1.250
X_3_	0.127	0.060	4.407	0.036*	1.135	1.008	1.278
Constant	-23.963	9.275	6.675	0.010	0.000		

X_1_: CD34^+^ cells; X_2_: CD133^+^ cell; X_3_: EPCs;

* *P* < 0.05.

**Figure 7.**
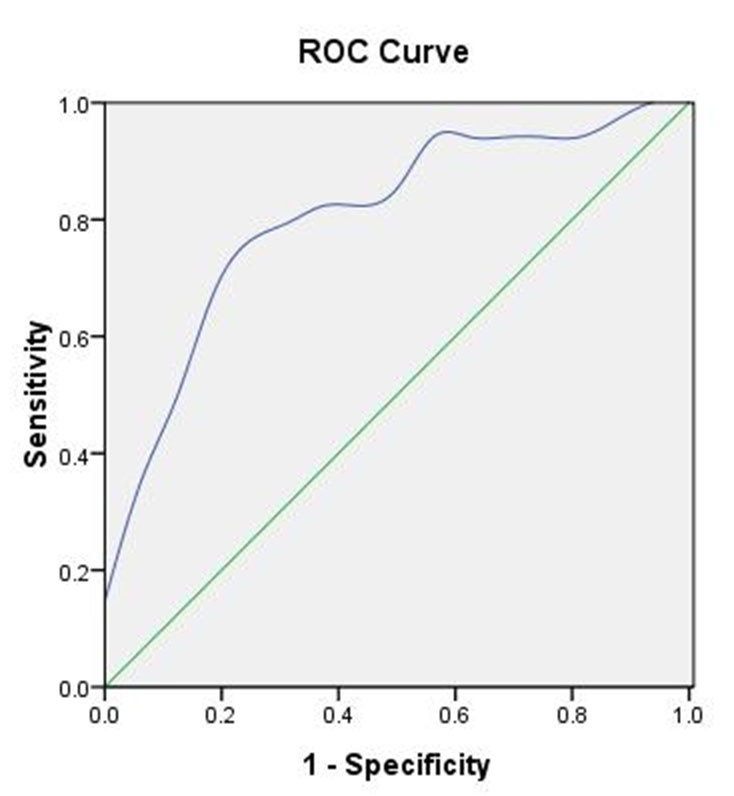
**Receiver operating characteristic (ROC) curve.** ROC created based on the percentage of CXCR4^+^/EPCs at day 7 after admission and the prognosis of patients with mild TBI. The area under the AUC was 0.807 (95% CI = 0.656-0.958, critical value = 42.35%, *p* = 0.003).

A correlation analysis between the level of CXCR^+^/EPCs at 7 days after TBI (Y-axis) and the prognosis state at 3 months after TBI (X-axis) was performed using Pearson correlation coefficients analysis. The data shows that the CXCR4^+^/EPCs level of TBI patients is correlated with the recovery of neurological function 3 months after TBI in all TBI patients (R= 0.605, P = 0.001), as well as in mild TBI patients (R = 0.518, P = 0.002). These findings further suggested that the level of CXCR4^+^/EPCs on day 7 was correlated with the patient’s prognosis. Patients with higher levels of CXCR4^+^/EPCs had poorer neurological outcome after TBI (Fig. 6).

A receiver operating characteristic (ROC) curve was made taking the percentage of CXCR4^+^ cells in EPCs as detection variable and prognosis at 3 months after TBI as state variable (Fig. 7, Table 3). The area under the curve (AUC) was 0.807 (95% CI: 0.656-0.958, P = 0.003) at a sensitivity of 0.840 and specificity of 0.727 for patients with mild TBI. It implied that AUC can be used as an indicator for predicting the prognosis of mild TBI patients. According to the ROC curve, the optimal cut-off value (the percentage of CXCR4^+^ cells in EPCs) is 42.35%, which could predict the prognosis of mild TBI, showing good application value. That is to say, when the percentage of CXCR4^+^ cells in EPCs in mild TBI patients at 7 days after injury was greater than 42.35%, a poor prognosis should be considered. As a result of prognostic follow up observation, 8 out of 27 mild TBI patients with good prognosis had percentages of CXCR4+ EPCs higher than 42.35% (false positive). On the other hand, 4 out of 30 mild TBI patients with poor prognosis showed a value lower than 42.35% (False negative).

**Figure 8.**
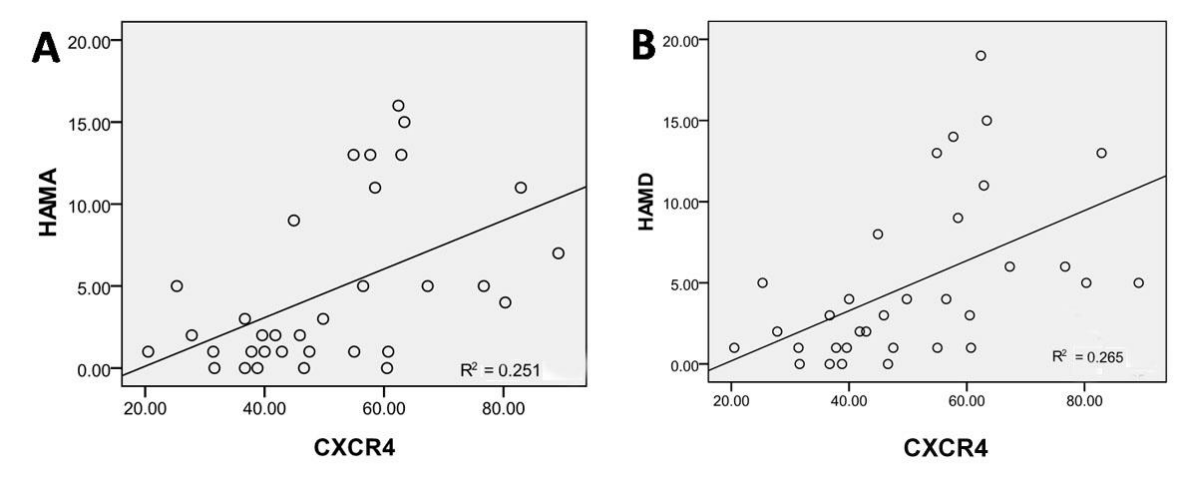
**Correlation between the percentage of CXCR4^+^/EPCs and psychological state after TBI. (A)** Figure shows significant correlation between the percentage of CXCR4^+^/EPCs at day 7 after TBI and the HAMA score at 3 months after discharge (R = 0.501, *p* = 0.003). **(B)** Figure shows significant relationship between the percentage of CXCR4^+^/EPCs at day 7 after TBI and the HAMD score at 3 months after discharge (R = 0.515, *p* = 0.002).

The percentage of CXCR4+/EPCs of both mild and moderate TBI patients at 7 days after TBI was positively correlated with the HAMA and HAMD scores at 3 months after discharge (HAMA: R = 0.501, P = 0.003; HAMD: R = 0.515, P = 0.002) (Fig. 8). It suggested that patients with higher percentage of CXCR4^+^ EPCs at 7 days after TBI presented obvious anxiety and depression.

**Table 3:** Parameters of receiver operating characteristic (ROC) curve created based on the percentage of CXCR4^+^ cells in EPCs at 7 days after admission and the prognosis of patients with mild TBI

Group	Critical value	Area under the curve	Standard error	*p*	95% confidence interval	
					Lower limit	Upper limit
Mild TBI (n = 57)	42.35	0.807	0.077	0.003*	0.656	0.958

* *p* < 0.05.

## DISCUSSION

Mild TBI primarily presented with headache, dizziness, and memory impairment, of which most which will be completely resolved within 1-3 months after TBI. However, a large proportion of mild TBI patients had emotional disturbances, which could last for 3-6 months after TBI. These patients are easily ignored by clinical physicians. Applying the GOS-E and ADLS at 3 months post-TBI, the 57 mild TBI patients could be subgrouped into good prognosis and poor prognosis groups. In order to explore the possibility of a mild TBI biomarker, we checked circulating EPC levels and its subtype of CXCR4^+^ EPCs in different subgroups of mild and moderate TBIs. To our knowledge, this is the first time anyone has shown a change in the circulating EPCs in mild TBI patients. It was surprising that both mild and moderate TBI increases circulating EPC, CD34^+^ and CD133^+^ cells levels compared to normal control in the early stage of TBI. There is no significant difference in the number of these cells between moderate TBI and mild TBI. Furthermore, changes in EPC levels (marked by double antibodies of CD34^+^/CD133^+^) did not show significant correlation with the prognosis of mild or moderate TBI. We also checked the change of the other subtypes of endothelial cells marked by CD34 and CD133. CD34^+^ cells are primarily detected on stem cells of endothelial lineage. CD133^+^ cells are highly expressed on EPCs at early stage and their changes were identified in severe TBI in our previous study [11,25]. EPCs participate in angiogenesis and maintain vascular endothelial stability, showing great significance for tissue repair [8, 9]. Under the condition of trauma and inflammation, partial EPCs could be mobilized from bone marrow to peripheral blood [26] and then to the sites of injury to participate in repair and regeneration of injured vessels. According to this and our previous studies, circulating EPCs are down-regulated in the early stage of TBI, and then up-regulated, no matter the severity of the brain injury. This seems to imply that inflammation can be induced by a TBI of any severity. In addition, changes in the EPC population could not be used to differentiate the TBIs of different severity.

This study also seems to show that the percentage of CXCR4^+^ cells in EPCs was significantly higher in the moderate TBI than the mild TBI patients from day 4 to day 21, and significantly higher in the poor prognosis group (group B) than the good prognosis group (group A) at day 7. At the same time, the level of the ligand of CXCR4, SDF-1α (CXCL12), a member of the CXC chemokine family, has been found to be higher in moderate TBI than mild TBI. SDF-1α has been shown to play a key role in migration of hematopoietic cells and tumor cells. It has also been shown to be excreted by nerve and glial cells, and plays an indispensable role in the occurrence and development of the nervous system [27]. CXCR4, as a specific receptor of SDF-1α, is expressed on the surface of nerve cells and glial cells in addition to monocytes and lymphocytes [28,29] in multiple regions including the caudate nucleus, globus pallidus and substantia nigra [30]. The synergism of SDF-1α and CXCR4 is crucial at the early stage of inflammation, where they act to control lymphocyte mobilization [31]. The inhibition of CXCR4 could significantly reduce the inflammation cytokines and cells [32]. There are several reports in the literature that confirm an inflammation reaction in mild TBI [33,34]. Thus, it is reasonable to imply that inflammation is induced in the both mild and moderate TBI.

The level of SDF-1α did not show significant difference between mild TBIs with either good or poor prognosis, while the CXCE4^+^ EPC levels could clearly differentiate between mild TBIs of different prognosis. Furthermore, several other studies have shown that injecting SDF-1α promoted the repair of injured brain tissue. However, this repair could be inhibited by blocking CXCR4 [35,36]. Thus, it is suggested that the expression of CXCR4 might be more sensitive in detecting brain injury, and a therapy targeting CXCR4 could be a new therapeutic strategy for TBI in the future.

Our previous study disused the increased expression of SDF-1α/CXCR4 in the injured brain induced angiogenesis and neural tissue repair [20]. CXCR4 is also expressed on the surface of EPCs [14, 15] and contributes to local angiogenesis and tissue reconstruction. Isabelle et al reported that SDF-1α promotes the homing and proliferation of EPCs and inhibits apoptosis of EPCs by binding to CXCR4 [19]. We found that serum SDF-1α was significantly increased in mild and moderate TBI patients compared to normal controls. However, moderate TBI patients have higher levels of serum SDF-1α than mild TBI patients in early stage (one and four days after TBI). It indicated that TBI stimulates SDF-1α secretion at an early stage after TBI, and SDF-1α release is correlated with the severity of brain injury.

Flow cytometry results show that during 1-4 days after TBI, the percentage of CXCR4^+^ cells in each cell population was significantly greater than the control group (P < 0.05), showing a similar tendency to serum SDF-1α level. The expression levels of SDF-1α/CXCR4 is consistent with the changes of circulating EPCs. These findings suggest that the SDF-1α/CXCR4 axis is likely to be one of factors that promote circulating EPCs and their subpopulation cells (CD34 and CD133) mobilization. In addition, high levels of SDF-1α/CXCR4 plays a chemoattractant role in leukocytes [37,38]. SDF-1α/CXCR4 axis is critical during allergic airway disease and potentially relevant in other inflammatory processes [39]. The role of the CXCR4^+^ EPCs in the inflammatory regulation in the TBI is strongly suggested. The specific biological mechanism on CXCR4^+^ EPCs after TBI is needed to confirm by further studies.

We found that the percentage of CXCR4^+^ cells in EPCs was significantly higher in moderate TBI than mild TBI patients, indicating that there may be a close relationship between the severity of TBI and its CXCR4^+^ EPC levels. The percentage of CXCR4^+^ cells in EPCs decreased closer to baseline sooner and more significantly in the mild TBI group than in the moderate TBI group. There was no significant difference between the mild TBI group and the baseline level of this index as early as 7 days after injury, while the moderate TBI group was still at a high level. The same trend was also found in the comparison between group A (good prognosis) and group B (poor prognosis). The percentage of CXCR4^+^ cells in EPCs quickly and significantly dropped in group A at 7 days after TBI, compared to group B. Therefore we hypothesize that the early reduction of this index indicates lighter injuries and better prognosis. However, there was on significant difference in the number of total circulating EPCs between the moderate and mild TBI patients.

Regression analysis shows that only the percentage of CXCR4^+^ cells in EPCs was correlated with the prognosis of patients with mild TBI (OR = 1.135, P = 0.036). We also use Pearson correlation analysis to prove that the percentage of CXCR4^+^ cells in EPCs on day 7 was correlated with TBI (mild and moderate) patient’s prognosis(R= 0.605, P = 0.000), even with significant relationship between group A and B (R = 0.518, P = 0.002). That is to say, with an increase in the percentage of CXCR4^+^ cells in EPCs, patient’s prognosis became poor. In addition, the higher the percentage of CXCR4^+^ cells in EPCs, the greater the HAMA (R= 0.501, P = 0.003) and HAMD (R = 0.515, P = 0.002) scores. These findings suggest that at 7 days after TBI, patients presented with a high percentage of CXCR4^+^ cells in EPCs and had significant symptoms of anxiety and depression. According to the ROC curve, AUC was 0.807 (95% CI: 0.656-0.958, P = 0.003) at a sensitivity of 0.840 and specificity of 0.727 for patients with mild TBI and the optimal cut-off value of the percentage of CXCR4^+^ cells in EPCs at 7days after TBI is 42.35%, confirming the diagnostic value of this index. It shows that this factor could be a valuable indicator to forecast a patients’ prognosis, even with mild TBI.

It is worth noting that there are limitations of this study such as relatively small sample size, which needs to be expanded, more moderate TBI patients and even severe TBI patients could be involved; and the follow-up time with the TBI patients should be extended further. In this study we found that high levels of circulating CXCR4^+^/EPC at 7 days after TBI is correlated with a poor prognosis after TBI. However, the mechanism is still not clear and worth investigating in a future study.

In conclusion, TBI stimulates SDF-1α/CXCR4 signaling activity and promotes circulating EPC mobilization. CXCR4^+^/EPC levels are correlated with poor prognosis after TBI. Therefore, circulating CXCR4^+^/EPC levels may predict the outcome in mild TBI patients. Targeting CXCR4^+^/EPC may also provide a novel therapeutic effect after TBI.

## References

[1] Eramudugolla R, Bielak AA, Bunce D, Easteal S, Cherbuin N, Anstey KJ (2014). Long-term cognitive correlates of traumatic brain injury across adulthood and interactions with APOE genotype, sex, and age cohorts. J Int Neuropsychol Soc, 20: 444-54 24670469 10.1017/S1355617714000174

[2] Falk AC, Alm A, Lindstrom V (2014). Has increased nursing competence in the ambulance services impacted on pre-hospital assessment and interventions in severe traumatic brain-injured patients? Scand J Trauma Resusc Emerg Med, 22: 20 24641814 10.1186/1757-7241-22-20 PMC3994652

[3] Pearson WS, Sugerman DE, McGuire LC, Coronado VG (20012). Emergency department visits for traumatic brain injury in older adults in the United States:2006-08. West J Emerg Med. 2012 8;13(3):289-93. 22928058 10.5811/westjem.2012.3.11559 PMC3426370

[4] Hou R, Moss-Morris R, Peveler R, et al (2012). When a minor head injury results in enduring symptoms: a prospective investigation of risk factors for postconcussional syndrome after mild traumatic brain injury. J Neurol Neurosurg Psychiatry, 83:217-23. 22028384 10.1136/jnnp-2011-300767

[5] Wilkinson CW, Pagulayan KF, Petrie EC, Mayer CL, Colasurdo EA, Shofer JB, et al (2012). High prevalence of chronic pituitary and target-organ hormone abnormalities after blast-related mild traumatic brain injury. Front Neurol, 3: 11 22347210 10.3389/fneur.2012.00011 PMC3273706

[6] Miller NR, Yasen AL, Maynard LF, Chou LS, Howell DR, Christie AD (2014). Acute and longitudinal changes in motor cortex function following mild traumatic brain injury. Brain Inj, 28: 1270-6 24841536 10.3109/02699052.2014.915987

[7] Tanriverdi F, Unluhizarci K, Kocyigit I, Tuna IS, Karaca Z, Durak AC, et al (2008). Brief communication: pituitary volume and function in competing and retired male boxers. Ann Intern Med, 148: 827-31 18519929 10.7326/0003-4819-148-11-200806030-00005

[8] Friedrich EB, Werner C, Walenta K, Bohm M, Scheller B (2009). Role of extracellular signal-regulated kinase for endothelial progenitor cell dysfunction in coronary artery disease. Basic Res Cardiol, 104: 613-20 19363666 10.1007/s00395-009-0022-6

[9] Zhang Y, Li Y, Wang S, Han Z, Huang X, Li S, et al (2013). Transplantation of expanded endothelial colony-forming cells improved outcomes of traumatic brain injury in a mouse model. J Surg Res, 185: 441-9 23953790 10.1016/j.jss.2013.05.073

[10] Liu L, Liu H, Jiao J, Liu H, Bergeron A, Dong JF, et al (2007). Changes in circulating human endothelial progenitor cells after brain injury. J Neurotrauma, 24: 936-43 17600511 10.1089/neu.2006.0250

[11] Liu L, Wei H, Chen F, Wang J, Dong JF, Zhang J (2011). Endothelial progenitor cells correlate with clinical outcome of traumatic brain injury. Crit Care Med, 39: 1760-5 21460712 10.1097/CCM.0b013e3182186cee PMC4915743

[12] Huang XT, Zhang YQ, Li SJ, Li SH, Tang Q, Wang ZT, et al (2013). Intracerebroventricular transplantation of ex vivo expanded endothelial colony-forming cells restores blood-brain barrier integrity and promotes angiogenesis of mice with traumatic brain injury. J Neurotrauma, 30: 2080-8 23957220 10.1089/neu.2013.2996 PMC3868401

[13] Bleul CC, Fuhlbrigge RC, Casasnovas JM, Aiuti A, Springer TA (1996). A highly efficacious lymphocyte chemoattractant, stromal cell-derived factor 1 (SDF-1). J Exp Med, 184: 1101-9 9064327 10.1084/jem.184.3.1101 PMC2192798

[14] Wang YB, Liu YF, Lu XT, Yan FF, Wang B, Bai WW, et al (2013). Rehmannia glutinosa extract activates endothelial progenitor cells in a rat model of myocardial infarction through a SDF-1 alpha/CXCR4 cascade. PLoS One, 8: e54303 23349848 10.1371/journal.pone.0054303 PMC3548813

[15] Fierro FA, Brenner S, Oelschlaegel U, Jacobi A, Knoth H, Ehninger G, et al (2009). Combining SDF-1/CXCR4 antagonism and chemotherapy in relapsed acute myeloid leukemia. Leukemia, 23: 393-6 18615106 10.1038/leu.2008.182

[16] Li LP, Kang JL, Xia W (2008). Effect of first, second, and third trimester placental factors on CD4, CCR5, and CXCR4 expression in human peripheral blood lymphocytes. Zhong Nan Da Xue Xue Bao Yi Xue Ban, 33: 461-7 18599991

[17] Gonzalez FJ, Carvajal MJ, Leiva L, Juarez C, Blanca M, Santamaria LF (1997). Expression of the cutaneous lymphocyte-associated antigen in circulating T cells in drug-allergic reactions. Int Arch Allergy Immunol, 113: 345-7 9130573 10.1159/000237597

[18] Banisadr G, Rostene W, Kitabgi P, Parsadaniantz SM (2005). Chemokines and brain functions. Curr Drug Targets Inflamm Allergy, 4: 387-99 16101548 10.2174/1568010054022097

[19] Petit I, Jin D, Rafii S (2007). The SDF-1-CXCR4 signaling pathway: a molecular hub modulating neo-angiogenesis. Trends Immunol, 28: 299-307 17560169 10.1016/j.it.2007.05.007 PMC2952492

[20] Li S, Wei M, Zhou Z, Wang B, Zhao X, Zhang J (2012). SDF-1alpha induces angiogenesis after traumatic brain injury. Brain Res, 1444: 76-86 22330724 10.1016/j.brainres.2011.12.055

[21] Ingebrigtsen T, Romner B, Kock-Jensen C (2000). Scandinavian guidelines for initial management of minimal, mild, and moderate head injuries. The Scandinavian Neurotrauma Committee. J Trauma, 48:760-766. 10780615 10.1097/00005373-200004000-00029

[22] Lawton MP, Brody EM (1969). Assessment of older people: self-maintaining and instrumental activities of daily living. Gerontologist, 9: 179-86 5349366

[23] Schretlen DJ, Shapiro AM (2003). A quantitative review of the effects of traumatic brain injury on cognitive functioning. Int Rev Psychiatry, 15: 341-9 15276955 10.1080/09540260310001606728

[24] Gyoneva S, Ransohoff RM (2015). Inflammatory reaction after traumatic brain injury: therapeutic potential of targeting cell-cell communication by chemokines. Trends Pharmacol Sci, 36: 471-80 25979813 10.1016/j.tips.2015.04.003 PMC4485943

[25] Li Z, Wang B, Kan Z, Zhang B, Yang Z, Chen J, et al (2012). Progesterone increases circulating endothelial progenitor cells and induces neural regeneration after traumatic brain injury in aged rats. J Neurotrauma, 29: 343-53 21534727 10.1089/neu.2011.1807 PMC3261789

[26] Trop S, Tremblay ML, Bourdeau A (2008). Modulation of bone marrow-derived endothelial progenitor cell activity by protein tyrosine phosphatases. Trends Cardiovasc Med, 18: 180-6 18790388 10.1016/j.tcm.2008.07.001

[27] Banisadr G, Fontanges P, Haour F, Kitabgi P, Rostene W, Melik Parsadaniantz S (2002). Neuroanatomical distribution of CXCR4 in adult rat brain and its localization in cholinergic and dopaminergic neurons. Eur J Neurosci, 16: 1661-71 12431218 10.1046/j.1460-9568.2002.02237.x

[28] Guyon A, Skrzydelsi D, Rovere C, Rostene W, Parsadaniantz SM, Nahon JL (2006). Stromal cell-derived factor-1alpha modulation of the excitability of rat substantia nigra dopaminergic neurones: presynaptic mechanisms. J Neurochem, 96: 1540-50 16476083 10.1111/j.1471-4159.2006.03659.x

[29] Banisadr G, Queraud-Lesaux F, Boutterin MC, Pelaprat D, Zalc B, Rostene W, et al (2002). Distribution, cellular localization and functional role of CCR2 chemokine receptors in adult rat brain. J Neurochem, 81: 257-69 12064472 10.1046/j.1471-4159.2002.00809.x

[30] Engelhardt B, Ransohoff RM (2012). Capture, crawl, cross: the T cell code to breach the blood-brain barriers. Trends Immunol, 33: 579-89 22926201 10.1016/j.it.2012.07.004

[31] Proudfoot AE, Uguccioni M (2016). Modulation of Chemokine Responses: Synergy and Cooperativity.Front Immunol,7:183. 27242790 10.3389/fimmu.2016.00183 PMC4871875

[32] Chen H, Xu X, Teng J, Cheng S, Bunjhoo H, Cao Y, Liu J, Xie J, Wang C, Xu Y, Xiong W (2016). CXCR4 inhibitor attenuates ovalbumin-induced airway inflammation and hyperresponsiveness by inhibiting Th17 and Tc17 cell immune response.Exp Ther Med,11(5):1865-1870 27168818 10.3892/etm.2016.3141 PMC4840799

[33] Bachstetter AD, Zhou Z, Rowe RK, Xing B, Goulding DS, Conley AN, Sompol P, Meier S, Abisambra JF, Lifshitz J, Watterson DM, Van Eldik LJ (2016). MW151 Inhibited IL-1β Levels after Traumatic Brain Injury with No Effect on Microglia Physiological Responses.PLoS One,11(2):e0149451. 26871438 10.1371/journal.pone.0149451 PMC4752278

[34] Yang SH, Gangidine M, Pritts TA, Goodman MD, Lentsch AB (2013). Interleukin 6 mediates neuroinflammation and motor coordination deficits after mild traumatic brain injury and brief hypoxia in mice.Shock,40(6):471-5. 24088994 10.1097/SHK.0000000000000037 PMC4218737

[35] Guyon A (2014). CXCL12 chemokine and GABA neurotransmitter systems crosstalk and their putative roles. Front Cell Neurosci, 5: 115 24808825 10.3389/fncel.2014.00115 PMC4009426

[36] Mao W, Yi X, Qin J, Tian M, Jin G (2014). CXCL12 inhibits cortical neuron apoptosis by increasing the ratio of Bcl-2/Bax after traumatic brain injury. Int J Neurosci, 124: 281-90 23984821 10.3109/00207454.2013.838236

[37] Ceradini DJ, Kulkarni AR, Callaghan MJ, Tepper OM, Bastidas N, Kleinman ME, et al (2004). Progenitor cell trafficking is regulated by hypoxic gradients through HIF-1 induction of SDF-1. Nat Med, 10: 858-64 15235597 10.1038/nm1075

[38] Kollet O, Shivtiel S, Chen YQ, Suriawinata J, Thung SN, Dabeva MD, et al (2003). HGF, SDF-1, and MMP-9 are involved in stress-induced human CD34+ stem cell recruitment to the liver. J Clin Invest, 112: 160-9 12865405 10.1172/JCI17902 PMC164291

[39] Gonzalo JA, Lloyd CM, Peled A, Delaney T, Coyle AJ, Gutierrez-Ramos JC (2000). Critical involvement of the chemotactic axis CXCR4/stromal cell-derived factor-1 alpha in the inflammatory component of allergic airway disease. J Immunol, 165: 499-508 10861089 10.4049/jimmunol.165.1.499

